# Antenatal physical activity: a qualitative study exploring women’s experiences and the acceptability of antenatal walking groups

**DOI:** 10.1186/s12884-016-0973-1

**Published:** 2016-07-22

**Authors:** Sinéad Currie, Cindy Gray, Ashley Shepherd, Rhona J. McInnes

**Affiliations:** Psychology, School of Natural Sciences, University of Stirling, Stirling, FK9 4LA UK; Institute of Health and Wellbeing, College of Social Sciences, University of Glasgow, 27 Bute Gardens, Glasgow, G12 8RS UK; School of Health Sciences, University of Stirling, Stirling, FK9 4LA UK

**Keywords:** Physical activity, Walking group, Public involvement, Antenatal care, Pregnancy

## Abstract

**Background:**

Regular physical activity (PA) can be beneficial to pregnant women, however, many women do not adhere to current PA guidelines during the antenatal period. Patient and public involvement is essential when designing antenatal PA interventions in order to uncover the reasons for non-adherence and non-engagement with the behaviour, as well as determining what type of intervention would be acceptable. The aim of this research was to explore women’s experiences of PA during a recent pregnancy, understand the barriers and determinants of antenatal PA and explore the acceptability of antenatal walking groups for further development.

**Methods:**

Seven focus groups were undertaken with women who had given birth within the past five years. Focus groups were transcribed and analysed using a grounded theory approach. Relevant and related behaviour change techniques (BCTs), which could be applied to future interventions, were identified using the BCT taxonomy.

**Results:**

Women’s opinions and experiences of PA during pregnancy were categorised into biological/physical (including tiredness and morning sickness), psychological (fear of harm to baby and self-confidence) and social/environmental issues (including access to facilities). Although antenatal walking groups did not appear popular, women identified some factors which could encourage attendance (e.g. childcare provision) and some which could discourage attendance (e.g. walking being boring). It was clear that the personality of the walk leader would be extremely important in encouraging women to join a walking group and keep attending. Behaviour change technique categories identified as potential intervention components included social support and comparison of outcomes (e.g. considering pros and cons of behaviour).

**Conclusions:**

Women’s experiences and views provided a range of considerations for future intervention development, including provision of childcare, involvement of a fun and engaging leader and a range of activities rather than just walking. These experiences and views relate closely to the Health Action Process Model which, along with BCTs, could be used to develop future interventions. The findings of this study emphasise the importance of involving the target population in intervention development and present the theoretical foundation for building an antenatal PA intervention to encourage women to be physically active throughout their pregnancies.

## Background

Physically active pregnant women reduce their risk of caesarean section [1], long term weight retention, obesity, chronic disease [2], pregnancy related discomfort [3] and gestational diabetes mellitus [4], whilst improving sleep quality [5], quality of life and feelings of happiness [6]. Consistent with research in the general population [7, 8], these benefits are most likely to be evident when pregnant women adhere to appropriate physical activity (PA) guidelines. Recently, the American College of Obstetricians and Gynecologists (ACOG) [9] reiterated that healthy women should be encouraged to be physically active throughout their pregnancies. Current recommendations within the UK also advocate active pregnancies and encourage healthy pregnant women with no contraindications to engage in at least 30 min of moderate intensity PA at least five times per week (where moderate intensity activity is defined as working ‘somewhat hard’ with a heart rate of between 125 and 155 beats per minute depending on the mothers age) [10].

Despite the numerous benefits and apparent recommendations, many pregnant women remain largely inactive. Research from Canada, USA, Ireland, Spain and Portugal estimate that less than 25 % of pregnant women met PA guidelines [11–14]. Furthermore, adherence to PA guidelines and engagement in any moderate intensity PA is seen to significantly decline throughout the course of pregnancy, further reducing the chances of pregnant women attaining the health benefits of an active lifestyle [13, 15–17].

This mismatch between recommendations and behaviour can be partially explained by investigating the barriers to engagement in PA throughout pregnancy. Past research demonstrates that women are aware of the benefits of being physically active during pregnancy including weight management, easier labour and improved or maintained fitness [16, 18, 19]. However, it is evident that the number and occurrence of perceived barriers outweigh the known and perceived benefits [18]. From a range of qualitative studies across the world, the main barrier to participation in antenatal PA is risk perception, where women are worried that engagement in PA will put their pregnancy and baby at risk [17, 18, 20–22]. This literature recognises other barriers such as lack of energy, sickness, being uncomfortable, little support from partners, families and friends, as well as lack of people to exercise/be active with.

A recognised determinant of PA in the general population is socioeconomic status (SES), where adults who live in deprived areas or have low SES are less likely to engage in PA or meet PA guidelines [23]. This association may have a negative impact on the short and long term health of pregnant women from low SES areas and their children. Previous studies investigating barriers and facilitators to antenatal PA have recruited diverse samples of pregnant women [17, 18, 20, 21], however, the experiences and facilitators of antenatal PA for women from deprived areas remain under-investigated. It is important to explore and understand the barriers to PA engagement for these women in order to adequately develop interventions relevant to their beliefs, barriers, facilitators and circumstances.

Many interventions have aimed to address PA in pregnant women, with differing success. These are often in the form of lifestyle interventions, with PA one of a multitude of health behaviours addressed [24]. Previous interventions have attempted to address known barriers to PA such as available facilities and social support, through group exercise classes [25], or to enhance knowledge through educational programmes [26]. However, there has been little research into what women feel would benefit them best and support their intention to be active throughout pregnancy.

Walking groups have become a popular intervention to promote PA in the adult (non-pregnant) population and have been found to significantly increase PA in adults [27], as well as in women during the postnatal period [28]. The group-based nature, alongside the inclusivity of walking may offer an antenatal intervention which addresses some of the barriers to PA engagement recognised by pregnant women.

Patient and public involvement in the development of health research is recognised at a national level [29]. Furthermore, involvement of patients during intervention design and development is recognised as an important and fundamental step to developing complex interventions [30]. Development of an intervention which is deemed acceptable by the target population should enhance engagement and therefore allow the intervention to be adequately tested with a representative sample. Since recruitment of a diverse sample is key to efficiently testing the effectiveness of an intervention, attaining the views and experiences of those who are least likely to attend (e.g. those from deprived areas) should provide a strong platform for the development and testing of an acceptable and intervention. Furthermore, by exploring women’s experiences and views of acceptability towards antenatal walking groups, we can identify the most appropriate and relevant factors which influence engagement in PA in this group and hence develop an intervention which is patient-led and acceptable.

In summary, existing literature investigates barriers to PA engagement in pregnant women, but the experiences of women, specifically from deprived areas, is lacking. Furthermore, there is very little research investigating what support and resources women find acceptable in relation to antenatal PA. The aim of this current study is to: explore women’s experiences of PA during a recent pregnancy; understand the barriers and determinants of antenatal PA; and explore the acceptability of antenatal walking groups.

## Methods

### Study design and participants

This qualitative exploratory study involved seven focus groups allowing participants to share their views and experiences. Participants were recruited through nurseries and mother-toddler groups from deprived areas of central Scotland (quintile one and two of the Scottish Index of Multiple Deprivation, SIMD [31]). Women who had at least one child born in the last four years were eligible to take part. Recruiting women with relatively recent pregnancies was a pragmatic decision driven by recruitment. Ethical approval was obtained from the University of Stirling, School of Health Sciences Research Ethics Committee.

### Data collection

Focus groups were facilitated by a member of the research team (CG) and each focus group (FG) lasted approximately one hour. The FGs took place in local nurseries and mother and toddler group premises. Participants were not provided with any incentives to participate. A semi-structured topic guide was developed specifically to explore women’s experiences of PA during pregnancy and their views on antenatal walking groups. All discussions were audio recorded using a digital recorder with informed consent from each participant.

The discussions were transcribed verbatim and the transcripts checked against the audio recording for accuracy. No individual names or identifying information were transcribed (pseudonyms were used as required). A grounded theory approach [32] was used to explore women’s experiences of antenatal PA and their views towards antenatal walking groups. An iterative process was used where themes arising in one interview were looked for in subsequent interviews, with new responses and themes arising added to the analysis, and previous interviews revisited to explore these. Analysis was conducted by two researchers (RM and AS) and consensus was reached on themes. In order to identify potential BCTs which could be applied to future interventions, the factors participants identified as increasing or decreasing their antenatal PA were categorised for each theme (SC). Relevant BCTs were then identified using the BCT taxonomy [33].

## Results

### Participants

A total of 24 women attended seven focus groups, with between two and five participants in each, between February and May 2011. Participants ranged in age and parity. Family size ranged from 1 to 6 children (mean = 2), with ages ranging from 6 weeks to 17 years (mean = 4.3 years). The age range of the youngest child in each family was 6 weeks to 48 months (mean = 16 months). Two women were pregnant at the time of the focus groups, and two women spoke English as a second language.

During analysis it became clear that women’s experiences of PA during pregnancy fell into three categories; physical/biological (pregnancy and physical wellbeing); psychological (thoughts and emotions) and social/environmental (community, time, childcare, health services). Hence, a bio-psycho-social framework was adopted to group the themes and present the data. Some women indicated that it was often difficult to move beyond the physical barriers related to PA during pregnancy, which were often perceived as insurmountable. However, deeper analysis revealed layers of influences on being active. This bio-psycho-social approach facilitates a holistic approach to understanding the complexity of influences and views towards PA in this population. The findings are presented using the women’s own words where possible as indicated by the use of quotation marks in the text or longer quotes. Longer extracts are labeled to indicate the Focus Group (FG1-7) and the participant (P1-5).

### Experiences relating to antenatal PA

The majority of women interviewed expressed a sense of having a busy/active lifestyle. For most, their daily activities related to walking: ‘walking everywhere’, ‘constantly being on the go’ and ‘running around after wee ones’. They described busy lives with multiple walks to and from nursery, school, the shops etc, and viewed housework as hard, tiring and sometimes seen as ‘exercise’. Being active for leisure or for fun was less apparent. While exercise was seen as ‘a good thing’, most women admitted becoming less active during their first and subsequent pregnancies (in comparison with pre-pregnancy activity levels) and while many said they planned to be active, this often just ‘didn’t happen’. Many women suggested that it is ‘ok to do what you have been doing before you are pregnant’ but ‘not to up the pace’ or to ‘start something new’ and several described fluctuations in their PA levels during pregnancy and between pregnancies. Already active women tended to keep active, but modified existing exercise regimes through ‘knowing your body’ or ‘listening to your body’ ‘just in case I’m injured or I damage the baby in some way’ rather than through any information and advice received. Many women also experienced a number of influences, as discussed below, which moderated their engagement with PA.

### Biological/physical issues

Most women recognised a number of benefits to being active during pregnancy such as getting ‘a better sleep at night because you’re knackered by it’ and generally ‘feeling better’. Some women mentioned having ‘an easier labour’, and there was recognition that it was beneficial to be fit, both for labour - ‘it’s not called labour for nothing’ - and to care for the baby after birth. However, some women reduced their PA through personal experience of having done too much and ‘suffering the next day’. The benefits were only cited as motivators by women who had directly experienced them as a result of being active during pregnancy. In contrast, women with no direct experience of any specific benefit appeared more skeptical. They described health messages regarding PA as ‘a con’ (‘aye they tell you that’), some had experienced a difficult labour despite being active and others suggested that being active ‘made labour harder’.

Some women who described being ‘constantly on the go’ or who felt ‘absolute exhaustion’ suggested that ‘even the thought of exercise can be a bit much’. Being pregnant with a growing foetus and number of pregnancy-related conditions, such as morning sickness, pelvic girdle pain (PGP) and pregnancy-related exhaustion were cited as barriers to being active.

*Some days when you are feeling sick in your first trimester*, *it’s quite like a…it’s quite difficult to make yourself go out and things when you’re not feeling well and at the end when you’re very tired as well. (FG5: P3)*

Weight loss or weight management, which is often considered to be a motivator for PA, was less apparent in the women’s dialogue. Where weight was mentioned, it was more often in the context of pregnancy allowing you to ‘eat a bit extra’ rather than a consideration of PA for reducing pregnancy weight gain. Some women who had been unable to lose weight gained in pregnancy appeared cynical about messages on weight management during pregnancy.

*“Apparently it makes it easier to shed the baby weight, that’s a myth by the way … That’s a myth, I’ve still no shed it four years later, that’s a myth. They do say that if you exercise when you’ve had a baby it makes it easier to shed, but that’s just a myth that is.” (FG1: P2)*

### Psychological issues

The psychological issues of being active during pregnancy included mood, stress, fear of harm, self-efficacy and self-confidence, as well as women’s beliefs about PA during pregnancy. Mood could act as both a motivator and de-motivator. Some women’s experience of improved mood or a ‘boost to your confidence’ following PA encouraged them to be active, even if they did not feel like doing so.

*I was up and about but I did feel better when I got out in fresh air and got out and saw people rather than sitting in the house*. (FG6: P4)

However, for other previously-active women, stopping their regular exercise/ PA during pregnancy had a depressing effect, which then demotivated them further.

Stress, related to busy lives juggling childcare, housework and jobs out of the home, motivated some women to seek ‘time out’ or exercise as a chance to ‘de-stress’, but for others it was a de-motivator where they considered they had ‘got enough exercise’ and just wanted to ‘crash on the couch once the kids go to bed’.

Fear of harm to either their baby (‘it’s so precious’) or themselves (‘your ligaments are all soft’) was a major influencing factor and was compounded by a lack of specific guidance or information to assist PA decision making. This incorporated exercise self-efficacy where women were not confident about engaging in PA or exercise due to fear of harm. While several agreed that some activities should be avoided, such as contact sports, ‘sit ups definitely’ and cycling ‘more for the risk of falling off’, there was little consensus on what types of activities are safe or on how much PA to do. Being ‘cautious’ pervaded the discussion on PA in pregnancy with the main emphasis being on ‘not overdoing it’.

*“I was pregnant with a second but I lost it and that’s because I was going to the gym a lot” (FG5: P5)*

In some cases, perceptions about what activities are acceptable changed in subsequent pregnancies. For some, a successful pregnancy might motivate increased PA but others, who reflected on being active throughout a first pregnancy, described limiting their PA in subsequent pregnancies as they realised how ‘precious’ their pregnancy was.

*I think you know what you can lose as well, you know? Second time around you know how wonderful they are so if you were to jeopardize that in any way by doing something stupid, that would, you know, you’d never be able to forgive yourself so* …(FG6: P3)

Low self-confidence, ‘shyness’ or the ‘risk of not knowing anyone’ prevented some women from taking part in organised PA.

*If you’ve got someone to do it with that’s maybe in the same situation, or even not, like at least if you’ve got somebody to go walking with or go swimming with. I always found that was easier, it motivated you more or… Like, if you’re on your own you’re less like to want to go …(FG3: P2)*

### Social/environmental issues

Social and environmental issues included meeting new people, and cultural, social or environmental influences such as accessibility of facilities and social attitudes, as well as influences from organisations such as the health services.

Social benefits of ‘getting out of the house’ and mixing with others especially if they were ‘bored sitting in their house’ were motivators for PA. ‘Time out’ away from the children ‘as much as you love them you need a little time without …’ and ‘enjoying a bit of me time’ were viewed as important, although this was sometimes moderated by feeling ‘guilty for leaving the house’ (FG6: P3).

Many women sourced their information from friends, family and occasionally via the internet, in addition to informing their own beliefs about risk and harm as discussed above. The negative attitudes of others put some women off.

‘*there’s a little too much focus on the fact that, you know, you’re pregnant, oh you can’t walk, you can’t…a very, kind of, can’t nature’ (FG4: P3).*

This negativity towards PA was also reflected in some of the information women received from health professionals such as midwives, physiotherapists and health visitors, which tended to be focused on ‘don’t do this, don’t do that, don’t do this’ and tended to make women less receptive to many health messages.

The structures of many of the women’s lives revealed a lack of time for doing PA for leisure.

*“No, because I’ve no time, by the time you get up in the morning and get them ready and get him to school and get the bairn to nursery and then it’s time to go back and pick him back up, and by the time you get him and give him his lunch and tidy up, it’s time to come back and pick him up from school, and then it’s dinner time and it’s bath time and then bedtime, the same routine, you’ve no got a minute.” (FG1: P3)*

Many of the women described the lack of accessibility of classes and PA groups. The timing of sessions were frequently inconvenient; for instance lunchtime sessions clashed with collecting children from nursery or toddler groups, early evening clashed with ‘tea-time’ and while classes later in the evening might suit some, lack of childcare could prevent participation for others. The location of classes also affected participation, as most of the mothers either did not have transport or did not wish to add travel time to the time out of family life required to attend classes.

Women’s experiences of health services described a focus on detecting abnormalities and monitoring physiological processes of pregnancy where contact with healthcare staff comprised ‘checks’ and lifestyle messages targeted at negative rather than positive behaviours.

*It’s just health that they go on about, isn’t it, like drinking, smoking but they won’t talk about exercise. (FG5: P2)*

However, when a midwife specifically recommended an activity, some women described being more determined to go.

*Yes because she said that so the minute I felt good, I thought, right I am going to do that and I remembered what she'd said. Whereas if she hadn't have pointed that out and I'd come across it in a leaflet, in amongst everything else I might not have. (FG2: P3)*

### Acceptability of antenatal walking groups

Participants gave a range of opinions about the acceptability of walking groups during pregnancy.

The positive social aspects of walking groups were highlighted, particularly for women who ‘don’t know anyone around here’ where this could potentially offer ‘… good company’ with ‘someone in the same situation as you’. However, for others, even the idea of an invitation to a walking group did not overcome the fear that they ‘may not know anyone’ or that ‘no-one might speak to you’. Although women didn’t ‘want[ing] to go out for a walk on your own’, the possible added advantage of an organised walking group ‘wouldn’t be worth it if you turned up on your own and you’ve nobody else. Or you turn up and you don’t know anybody and it’s awkward’. Not being a ‘joiner-inner’ also affected participation.

*I think, does it not also depend on the confidence of the people. If you’re not a joiner-inner then you’re not going to do that. Because I think sometimes you’re at your most vulnerable when you’re pregnant, especially the first time, because you don’t know what’s happening, so that will have an impact on whether you’re going to go and join a group or something. (FG6:P2).*

Some suggested that meeting up first, for example, for coffee, might help.

*Like you say, either tea or coffee, whatever, and then go, right, go on, you’ve had your lazy time, come on, we’re going for a walk, you’ve all met each other, been introduced and that, now it’s time to …(FG5: P5).*

Walking did not appear to be the most popular activity, as women generally wanted activities that made them feel better, for fun and to ‘relax’. While walking was viewed as acceptable and beneficial by some women, for others it was not leisure but a mode of transport and often ‘stressful’, as it related to nursery and childcare schedules. Walking was often viewed as functional or ordinary: ‘why would you [go on a walk]’, especially for those who ‘can walk anytime’ as part of their lifestyle and several said they *‘*would lose interest after a couple of times if it was just walking all the time’ and they would ‘find it boring’

Women were very clear that the choice of group facilitator was important and that this should be someone ‘who knows you’ and is ‘interested in you’ and your family, who is ‘dynamic’, ‘motivates you’ and ‘makes it fun’. Personality rather than professional background was more important, and women suggested that the ideal leader would be a fun person who cared, had some first aid experience and probably had children themselves. A discussion regarding who may be appropriate to lead walking groups indicated that groups run by midwives did not appeal to women. Women thought that leading walking groups was not midwives’ ‘kind of work’ and that a midwife would be ‘judging you’. For some, having a midwife leading the walks would definitely be off-putting, as they would have to ‘watch what they said, did or ate’. Many women would prefer someone with whom they would feel more comfortable.

*When you’ve got to do things and you’ve got a midwife standing in front of you, it’s not as if you can just…well if it was me, as I say, I would feel so feared to do anything in case she was thinking, well…but if it’s just somebody normal, I’m not saying they’re not normal but just somebody that’s like us, then you could just have an adult conversation with them, tell them this, they tell you that and just sit and have a wee laugh or something like that but, no, I wouldn’t like it if it was a midwife but…(FG4: P2)*

Some women discussed being discouraged from walking groups due to not wanting to make a commitment to attend regularly as it ‘depends on your mood’, ‘you might wake up and just not feel like it’ plus the ‘weather could be awful’ and ‘that would put you off’.

The women were also asked for suggestions on how to encourage participation in walking groups and other PA interventions during pregnancy. They suggested that some of the concerns relating to health and wellbeing could be overcome by provision of relevant information from a trusted source delivered face to face at the appropriate time. Women expressed that they would be most receptive to information on PA during the second trimester of pregnancy, ‘once the morning sickness has gone’ and before they ‘get too big’ although most women noted that they did not have antenatal appointments at this time. Furthermore, antenatal care in this population did not allow ‘time for health discussions’ ‘only time for checks’. A lack of specific information or guidance in how to manage pregnancy related conditions left some women unsure if exercise may be helpful.

*I don’t know, when I got told that I basically couldn’t do nothing [due to pelvic girdle pain], my life stopped. The only time I exercised is when I walked from the taxi to the physio department and back, that was the only time I really done anything. … they didn’t tell me if there was stuff I could actually do, so basically I sat and ate and that was it, sat on the couch and ate with the feet up. What else was I meant to do?” (FG1: P2)*

Psychological issues emerged as a key barrier to pregnant women engaging in organised antenatal PA programmes but women suggested various ways to overcome this. For instance, ‘shyness’ or the ‘risk of not knowing anyone’ could be overcome by receiving a personal invitation (from their midwife or class facilitator) or being able to bring a friend. Some felt that this support may only be needed at the start to enable them to ‘just turn up’; others felt that it may continue to act as a motivator in keeping them going.

Some women indicated that participation may be improved by scheduling groups or classes to fit in with nursery/ school times and by providing evening options for women who work or rely on a working partner. The provision of a crèche may help some women, although others were unsure of using such facilities: they expressed concerns about the safety of their children, about trusting those working there and their own guilt for using a crèche so that they could have fun. For some being able to ‘go to with the kids*’* may improve access through removing the requirement for childcare and may have the added bonus of being able to ‘help to break the ice’.

For the small number of women who were ‘not interested, to be honest’ or ‘can’t be bothered’ with exercise or who found exercise ‘boring’, the choice of activity could be especially important. Activities such as swimming or Zumba were considered to be more enjoyable than walking groups. Women described how ‘great it [swimming] feels’ and enjoyed the sense of ‘weightlessness’ and might choose to do yoga to ‘de-stress’ and ‘relax’ and Zumba ‘to have a laugh’.

Finally, information about locally available activities could be better facilitated. During the discussions it emerged that in addition to there being limited classes for pregnant women, there was also a lack of information about classes that did exist. Much of the information is passed ‘by word of mouth’, but women expecting their first baby were not linked into this social and informational network and also did not access nurseries and toddler groups where such classes might be advertised. Some women suggested they would welcome a leaflet listing local classes specifically for pregnant women, safe walk routes and the location of toilets.

### Behaviour change techniques for future antenatal PA interventions

The emerging themes and extracts of data provide indication of potential BCTs which could be applied in future interventions. These BCTs and examples are summarised in Table 1. In addition to these BCTs, involving children (childcare) and ensuring a fun and relaxing environment would be recommended.

**Table 1:** Factors influencing participation in PA in pregnancy

	Increase engagement with PA	Decrease engagement with PA	BCTs which could be employed into an intervention (BCT taxonomy identifier [33])	Potential interventions to increase PA
Biological/physical	− Good information from a trusted source − Personal experience of the benefits	− Pain, discomfort − Pregnancy size − Sickness − Tiredness	− Information from a credible source (9.1) − Overcome physiological barriers through problem solving (1.2) − considering pros and cons (9.2) − Enhance experience of benefits through self-monitoring of outcomes of behaviour (2.4) − Focus on past success (15.3)	− Individually tailored information (specifically related to pregnancy challenges e.g. PGP) − Taster sessions to facilitate personal experience of benefits
Psychological	− Experience of feeling good / wellbeing after PA − Having fun − De-stress/relax	− Can’t be bothered, effort − Lack of confidence / self-esteem − Fear of harm	− Encourage reflections of wellbeing after PA with self-monitoring of outcomes of behaviour (2.4) − Enhance peer support and confidence through social support: practical and emotional (3.2, 3.3) − Address fear of harm through instruction on how to perform behaviour (4.1) − demonstration of the behaviour (6.1) − information about health consequences (5.1)	− Classes which provide opportunities for fun and relaxation − Personal invitation / peer support − Specific classes for pregnant women − Tailored information linking classes with health services
Social/ environmental	− Supportive partner − Meeting others	− Childcare − Time − Lack of information − Lack of advice from midwives/Health services	− Facilitate meeting others and support through social support: practical and emotional (3.2, 3.3) − Encouraging awareness of support from others through information about others approval (6.3) − Additional information from midwives through information about health consequences (5.1) − instruction on how to perform behaviour (4.1) − credible source (9.1) − Overcoming barriers such as childcare through problem solving (1.2)	− Social opportunities alongside classes/groups − Involving children/childcare

## Discussion

Women’s experiences of PA during pregnancy evolved into three themes: biological/physical, psychological and social/environmental. Furthermore, women identified a range of factors which should be considered when developing, implementing and evaluating PA interventions, including antenatal walking groups, alongside factors and considerations which would enhance the acceptability of such a group.

Risk perceptions and fear of safety when engaging in antenatal PA were commonly cited barriers to PA participation. These concerns correspond with literature recognising risk as a major determinant of PA cessation in pregnant women [20], and could be explained by considering the PA advice and support women are offered in usual antenatal care. It is recognised that PA is not a priority for health professionals providing antenatal care as there are many other important health messages and information to be delivered during routine visits [34, 35]. When information or advice on PA is provided, this is often vague [36], which may leave women wary of undertaking PA (particularly new types of activities) due to unaddressed fears. Women who obtain or read antenatal PA guidelines from governing bodies such as the Royal College of Obstetricians and Gynaecologists (RCOG) [10] or ACOG [9], may notice that much of the advice is negative and often unclear, and mainly focuses on activities to avoid and complications to be aware of rather than focusing on the safe activities and benefits which pregnant women may experience. Emphasis on speaking to a GP or health professional prior to engagement in PA may further accentuate risk perceptions of PA engagement, especially when these health professionals are not forthcoming with PA-related information or advice. Although the guidelines are accurate and most women should seek medical advice if beginning a new PA regime when pregnant, the guidelines need to be supplemented with conversations, personal contact and support to ensure women understand them and are encouraged to engage in regular PA. Personal contact and support from health professionals to supplement printed recommendations may be important for facilitating PA in pregnant women, and lack of such support was recognised by the participants of this study as a barrier to PA engagement. Research indicates that some antenatal care providers feel confident in delivering this information, however, they feel that they do not have the time and resources to do so [35]. These findings, along with the existing literature, support the case for additional resources and time for health care professionals to provide advice and information to pregnant women in order to enhance PA engagement.

In the focus groups, women identified some potential facilitators for engagement in PA when pregnant, including many psychological and social factors such as self-efficacy, social support and risk perceptions. The importance of self-efficacy is recognised as a significant predictor of antenatal PA [37], hence ensuring that self-efficacy for PA is enhanced in pregnant women is essential to encourage participation. The findings also indicate that social support through meeting others and having supportive partners was a facilitator of PA. Feeling supported and sharing experiences of pregnancy and antenatal PA is recognised as an important motivator for women [38], and this is an important factor to consider when developing antenatal PA interventions. These constructs, beliefs, barriers and facilitators directly relate to a range of BCTs [33] which can be incorporated into an intervention. An example of relevant BCTs related to each barrier and facilitator is presented in Table 1. These could be adapted for future antenatal PA interventions.

Given the range of social cognitive factors involved in PA behaviour within this group of women, application of a social cognitive theory may help to structure any future intervention [39]. One such theory which could be deemed appropriate is the Health Action Process Approach (HAPA) [40]. The HAPA recognises a range of constructs which are influential in developing intention to perform a behaviour (risk perceptions, outcome expectancies, self-efficacy), translating that intention into action (action and coping planning, self-efficacy, barriers and resources including social support) and maintaining the behaviour (action control, self-efficacy, barriers and resources including social support). Application of the HAPA in the development of future interventions through addressing risk perceptions, outcome expectancies and self-efficacy, may be effective in addressing the barriers and enhancing the facilitators recognised by participants.

Women responded rather negatively to the idea of walking groups, but provided a range of logistical issues which may improve their acceptability. The importance of the choice of the potential walking group leader was recognised by participants. It was clear that women wanted fun and enjoyable sessions, and felt that a midwife may judge or intimidate them. Delivery of an intervention by a non-maternity health professional may reduce this preconception of judgement. However, this finding contradicts the desire for expert advice which also women requested. A review of walking groups for health by Kassaovu et al. [27] found no difference in PA between groups run by health professionals and groups run by trained walk leaders, therefore these two facets (expert advice vs fun, engaging staff) may need to be recognised as two distinct features of an intervention.

Timing of the group was also considered by participants to be important, specifically in relation to women with children who felt that they needed childcare or transport in order to attend a group at a certain time. Walking groups for postnatal women have been successful in the past, where issues of childcare have been addressed through development of buggy walks [28]. Inclusion of childcare or involvement of children in PA activities may encourage participation and enhance acceptability. Many women mentioned that they would prefer a variety of activities rather than just walking. Although walking is recognised as the most popular, accessible and common PA by pregnant women [17, 41] many of the women in this study perceived walking as a form of transport rather than an enjoyable leisure activity, and felt that they did not need to attend an organised group to walk. Understanding and capitalising on the motivations women have for attending walking groups or engaging in PA is important as some women saw walking groups as a place to socialise and have fun, rather than to help them be more active. This motivation and potential mismatch between participant expectancies and intervention aims should be recognised and incorporated to ensure women enjoy classes, which will, in turn, promote engagement and thereby potential success enhancing PA.

Finally, recruitment to a PA intervention or walking group was considered by participants. Many women mentioned they were bombarded with information in the first antenatal appointment. Hence, recruitment to such interventions requires development of a personal connection with researchers or intervention/class leaders. Research indicates that under study conditions, a dedicated midwife who spends time with women explaining a PA intervention enhances understanding and therefore participation however this may need to be adapted for incorporation into routine antenatal care [42].

Participants described a range of barriers and facilitators to antenatal PA, as well as some logistical issues which should be considered when developing a walking group or PA intervention for pregnant women. As it stands, a walking group does not seem acceptable to women living in deprived areas due to its general lack of appeal and a range of logistical and methodological issues such as child care, timing and resources. Addressing these factors along with inclusion of theory, such as the HAPA, may help to engage and attract women to such interventions, therefore improving PA behaviour and subsequent outcomes.

Although this exploratory qualitative research provides insights into women’s views towards antenatal PA and offers considerations for future intervention development, there are some limitations which should be noted. The sample of women who participated in the focus groups were from deprived areas of Scotland, their views and needs for intervention may differ to those in other countries or areas of lesser deprivation. Furthermore, there was diversity in the amount of time since participants had been pregnant, ranging from zero to four years. Although all women were discussing their experiences and views retrospectively, this varying time frame between participants have influenced their opinions, and may have contributed to some differences in viewpoints. However, research indicates that after 20 years, women accurately recall health behaviours related to their pregnancy [43].

## Conclusions

This study explores socio-economically disadvantaged women’s views and experiences of PA during pregnancy. The participants identified many barriers to PA in pregnancy; mainly risk perceptions but at the same time described potential facilitators such as self-efficacy and social support, including childcare. Women also highlighted opportunities for fun and relaxation as drivers for participation. These experiences and views relate closely to the HAPA, which along with BCTs, could be used to inform the development of future interventions.

Antenatal walking groups do not seem acceptable in their traditional format, as walking was seen as boring and not classed as exercise. There needs to be some adaptions for this group of women, specifically in relation to the types of activity offered, and the differing motivations for attendance and focus between women and researchers/health professionals (for example, fun versus behaviour change). Inclusion of aerobic or dance activities as well as/instead of walking may encourage engagement. This research emphasises the importance of attaining the views of the targeted population for theoretical and logistical development, engagement and acceptability of PA interventions.

## Abbreviations

ACOG, American College of Obstetricians and Gynaecologists; BCT, behaviour change technique; FG, focus groups; HAPA, health action process approach; P, participant; PA, physical activity; PGP, pelvic girdle pain; RCOG, Royal College of Obstetricians and Gynaecologists; SES, socio-economic status; SIMD, Scottish Index of Multiple Deprivation
